# Transcription Factor p53 Suppresses Tumor Growth by Prompting Pyroptosis in Non-Small-Cell Lung Cancer

**DOI:** 10.1155/2019/8746895

**Published:** 2019-10-13

**Authors:** Tianze Zhang, Yongchao Li, Ruidong Zhu, Pengcheng Song, Youlei Wei, Tian Liang, Guangquan Xu

**Affiliations:** ^1^Department of Thoracic Surgery, The 2nd Affiliated Hospital of Harbin Medical University, Harbin 150081, China; ^2^Department of Obstetrics and Gynaecology, The 1st Affiliated Hospital of Harbin Medical University, Harbin 150081, China

## Abstract

**Objective:**

To evaluate the effect of p53 on pyroptosis and its inhibitory role on tumor growth in non-small-cell lung cancer (NSCLC).

**Methods:**

The correlation of p53 and pyroptosis was determined in tumor tissues of NSCLC patients. The pyroptotic level was detected in A549 cells to clarify the effect of p53 on pyroptosis. p53 overexpression A549 tumor-bearing mice were used to clarify the therapeutic target of p53 in NSCLC treatment.

**Results:**

p53 expression level was positively related to pyroptosis in NSCLC tissues. In *in vitro* assays, p53 directly regulated pyroptosis in A549 cells. p53-specific knockdown blocked lipopolysaccharide- (LPS-) induced pyroptosis. In *in vivo* assays, p53 overexpression in A549 markedly decreased tumor growth and death rate by increasing the pyroptotic level.

**Conclusions:**

Upregulation of p53 prompts pyroptosis to produce anti-NSCLC effects suggesting the potential of p53 on suppressing tumor growth in NSCLC patients.

## 1. Introduction

Non-small-cell lung cancer (NSCLC) is the most life-threatening neoplasm, which is generally diagnosed at the advanced stage with low 5-year survival rate [1]. Canonical treatments include chemotherapy and targeted drug therapy to prolong patients' survival and relieve symptoms during NSCLC [2]. Approximately 80% of lung cancer is attributed to NSCLC, and its increasing mortality and morbidity heighten the need for advanced treatments.

Researches focused on programmed cell death have received considerable attentions [3]. Apoptosis has been instrumental in our understanding of programmed cell death, which happens to defend impaired stimulus and control deteriorated accumulations. The development of apoptosis is related to the formation of apoptotic bodies and the activation of caspases [4, 5]. Recently, there has been renewed interest in a noncanonical programmed cell death: pyroptosis, which is related to infectious, spontaneously inflammations, and autoimmune conditions [6]. In contrast, there is much less information about the effects of pyroptosis. As a foreign composition in an organism, NSCLC cells predispose to initiate an autoimmune response and in turn trigger a series of inflammatory cascade [7]. Given these evidence, we thereby hypothesized that pyroptosis possesses a tight correlation with the progression of NSCLC.

Tumor suppressor gene, p53, is considered to suppress cell carcinogenesis via regulating cell cycle [8]. Previous studies have demonstrated that the mutation of p53 not only loses the function of tumor suppressor but also regains the function of oncogene to promote malignant transformation of cells [9]. Among several noncanonical cell death processes, including apoptosis, ferroptosis, necroptosis, and paraptosis, p53 is the most important factor to switch these progression via regulating multiple signaling pathways [10]. In addition, it is reported that p53 is tightly related to the occurrence and progression of NSCLC, with underinvestigated underlying mechanisms remained [11]. Here, we hypothesized that p53 is the key mediator to pyroptosis exerting the beneficial effects on tumor growth inhibition to improve the survival as well to slow the development during NSCLC.

## 2. Materials and Methods

### 2.1. Human Subjects and Informed Consent

NSCLC patients that were newly diagnosed received treatment at the Second Affiliated Hospital of Harbin Medical University. The study was approved by the Institutional Ethical Board, and tumor samples were collected upon a written informed consent. Tumor samples were extracted, and p53 and pyroptotic mRNA levels were analyzed by real-time PCR analysis.

### 2.2. Animals

The protocols of animal experiment were in agreement with the Ethics Committees of Harbin Medical University. Experimental principles were fulfilled with the *Guide for the Care and Use of Laboratory Animals* published by the US National Institutes of Health (NIH Publication No. 85-23, revised 1996). Six-week-old BALB/c nude mice were provided by the Animal Center of the 2nd Affiliated Hospital of Harbin Medical University (Harbin, China) and housed in a dedicated room with 12 h dark/light cycle, controlled temperature (22 ± 1°C), and constant humidity (55 ± 5%). After 1 week acclimatization, thirty nude mice bearing A549 tumor xenografts were divided into two groups randomly: controls and p53 overexpression group. The volumes of the tumors were measured every week for 2 months.

### 2.3. Cell Culture and Treatment

A549 cell lines were obtained from Shanghai Institutes for Biological Sciences (SIBS, China) and cultured in RPMI-1640 medium (Thermo Fisher Scientific, Waltham, MA, USA) supplied with 10% fetal bovine serum (FBS, Gibco, USA) and 1% penicillin/streptomycin (100 *μ*g/mL) in 5% CO_2_ at 37°C. Before treatment, cells were incubated with FBS-free restriction/treatment medium overnight.

### 2.4. Cell Transfection

A549 cells were seeded into a 6-well plate at a density of 2 × 10^6^ cells/well. At the confluence of 80%, cells were starved in a serum-free medium for 24 h before transfection and then transfected with 100 nM pcDNA3.1-p53 plasmid using Lipofect2000 transfection reagent according to the manufacturer's instructions, pcDNA3.1 treatment as control. The transfection medium was replaced by regular growth medium after 6 h transfection.

### 2.5. Real-Time PCR

Total RNA was harvested from cells using TRIzol reagent (Invitrogen, Carlsbad, CA, USA) according to previous studies [12]. cDNA synthesis was performed using a High-Capacity cDNA Reverse Transcription Kit (Applied Biosystems, Carlsbad, CA, USA; Cat. no. 4368814) according to the manufacturer's instructions. The levels of p53 and pyroptotic factors, including caspase-1, cleaved caspase-1, apoptosis-associated speck-like protein containing CARD (ASC), and nucleotide-binding domain and leucine-rich repeat-containing 3 (NLRP3), were determined by SYBR Green I incorporation method and ABI 7500 fast real-time PCR system (Applied Biosystems, USA). GAPDH was used as internal control.

### 2.6. Western Blotting

Western blotting analysis was performed as previously described [13]. Protein samples were extracted with RIPA buffer supplemented with protease inhibitors and quantified using the BCA method (Beyotime, Shanghai, China). Protein samples were fractionated by SDS-PAGE (10% polyacrylamide gels) and transferred to a nitrocellulose membrane. After blocking with 5% nonfat milk, the membrane was incubated with the primary antibodies for p53, caspase-1, cleaved caspase-1, NLRP3 (Wanlei Bio, Shenyang, China), and ASC (Absin Bioscience Inc., Shanghai) followed by incubation with a florescence-labeled secondary antibody. The blotted proteins were detected and quantified with an Odyssey Infrared Imaging System (LI-COR, Lincoln, NB, USA). GAPDH (Santa Cruz Biotechnology, Dallas, Texas) was used as internal control.

### 2.7. Immunofluorescent Staining

Immunofluorescent staining was performed as previously described [14]. Cells were fixed with 4% paraformaldehyde for 30 min at room temperature. 0.4% Triton X-100 was used for penetration and sealed by normal goat serum for 2 h at 37°C. p53 and NLRP3 were stained with primary antibody for 24 h at 4°C. Then, the cells were probed with florescence-labeled secondary antibody and DAPI for nuclei, observed under a confocal laser scanning microscope (FV300, Olympus, Japan).

### 2.8. Proliferation Assay

A549 cells were seeded in a 96-well plate. At an indicated time point, CCK-8 reagent was added to each well (10 *μ*L/well) and continually incubated for 4 h. Then, the optical density (OD) value (450 nm) was determined by an enzyme-linked immunosorbent assay plate reader (Bioreader).

### 2.9. Statistical Analysis

All values were presented as mean ± S. E. M. Statistical comparisons were performed by Student's *t*-test between two groups or one-way ANOVA for multiple comparisons. *p* < 0.05 was considered to indicate a significant difference. Data were analyzed using the GraphPad Prism 5.0 software. Correlations between p53 and pyroptosis were assessed by using Pearson, Spearman, and Kendall's rank correlation coefficient analyses [15].

## 3. Result

### 3.1. p53 Expression Level Is Positively Related to Pyroptosis in NSCLC Tissues

**Figure 1:**
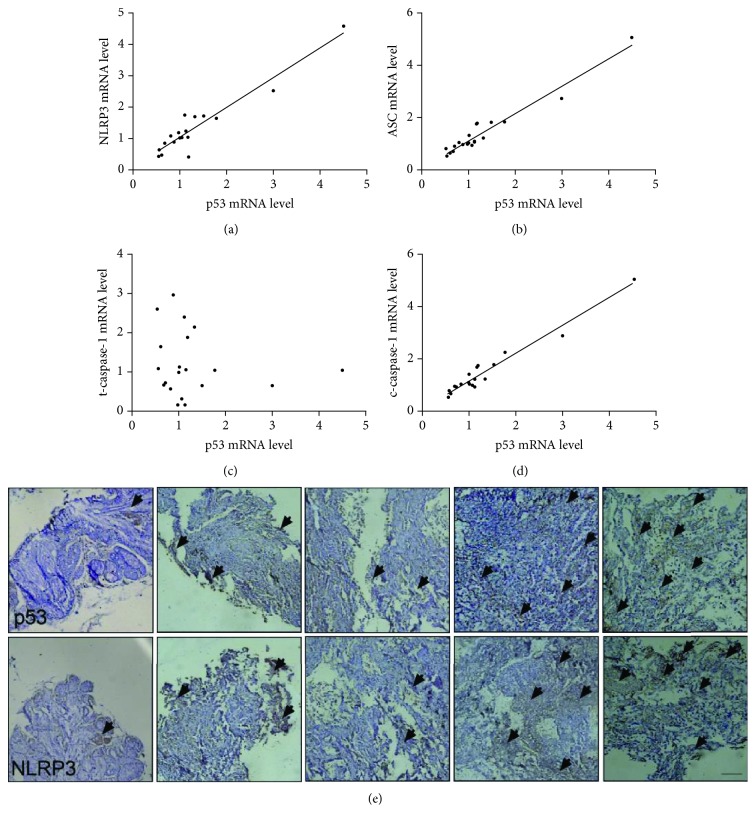
p53 is positively related to pyroptosis in the tumor tissue of NSCLC patients. (a–d) mRNA levels of NLRP3, ASC, caspase-1, and cleaved caspase-1 were detected by real-time PCR and analyzed by SPSS. Wild-type *p53* mRNA level shows a positive correlation with NLRP3, ASC, and cleaved caspase-1 in tumor tissues of 20 NSCLC patients. (e) p53 protein level (upper panel) and NLRP3 (lower panel) expression were determined by DAB staining. Arrows represent DAB-positive staining area. Scale bar equals 50 *μ*m.

The main process of pyroptosis includes the activation of caspase-1 and formation of inflammasome, and the network is comprised of cleaved caspase-1, ASC, and NLRP3 [16]. To assess the correlation between p53 and pyroptosis in the tumor tissue of NSCLC patients, we analyzed the data from canonical correlation analyses (Pearson, Spearman, and Kendall). The results indicate that the p53 mRNA level positively correlated with pyroptosis (Figures 1(a)–1(d)). Statistical data (Table 1) digitalize the correlation, providing a strong evidence for the involvement of p53 in pyroptosis. Since p53 is a transcription factor that acts upon its direct target genes, it is necessary to determine the protein level. The correlation analyses of the p53 protein level with pyroptosis are consistent with the p53 mRNA level (Figure 1(e) and [Supplementary-material supplementary-material-1]). Thus, we concluded that p53 level is positively related to pyroptosis, implying the inhibitory potential of p53 on NSCLC by pyroptosis.

### 3.2. p53 Is Responsible for Pyroptosis in A549 Lung Cancer Cells

**Figure 2:**
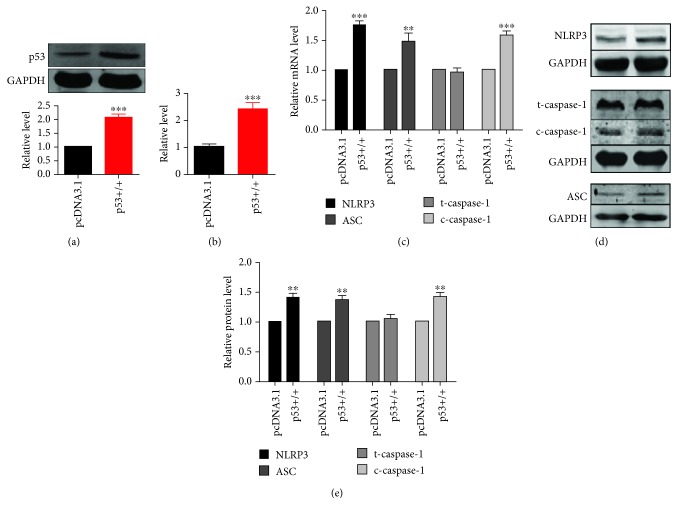
Overexpression of p53 elevates pyroptotic level in A549 NSCLC cells. A549 lung cancer cells were transfected with p53 overexpression or negative control vector (pcDNA3.1). (a) p53 protein expression level was determined by western blotting analysis; *n* = 5, ^∗∗∗^*p* < 0.001. (b) *p53* mRNA level was determined by real-time PCR; *n* = 5, ^∗∗∗^*p* < 0.001. (c) Pyroptotic factor mRNA levels were determined by real-time PCR; *n* = 7, ^∗∗^*p* < 0.01, ^∗∗∗^*p* < 0.001 versus the negative control group. (d, e) Pyroptotic proteins were determined by western blotting, and statistical results were obtained by densitometric analysis and normalization to GAPDH; *n* = 5, ^∗∗^*p* < 0.01.

p53 is generally considered as a transcription factor to regulate several programmed cell deaths [10]. To further verify the involvement of p53 in pyroptosis during NSCLC, A549 cells were transfected with p53 overexpression vector or pcDNA3.1 negative control vector. Results of transfection efficiency were assessed, illustrating that the transfection of pcDNA3.1-p53 significantly elevated p53 protein and mRNA levels (Figures 2(a) and 2(b)). Overexpression of p53 increased pyroptotic level, including the upregulation of NLRP3, ASC, and cleaved caspase-1 both in mRNA and protein levels (Figures 2(c)–2(e)). Additionally, p53 agonist was used to activate p53 and similar results were obtained that activation of p53 prompted pyroptosis in A549 cells ([Supplementary-material supplementary-material-1]).

**Figure 3:**
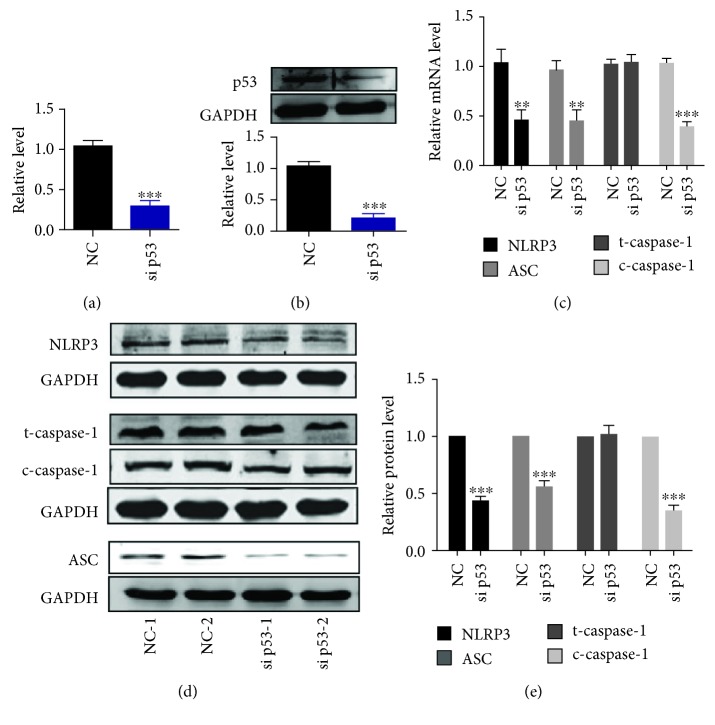
Knockdown of p53 inhibits pyroptotic level in A549 NSCLC cells. (a) *p53* mRNA level was determined by real-time PCR. A549 lung cancer cells were transfected with siRNA p53 or negative control vector (NC); *n* = 6, ^∗∗∗^*p* < 0.001. (b) p53 protein expression level was determined by western blotting analysis; *n* = 5, ^∗∗∗^*p* < 0.001. (c) Pyroptotic factor mRNA levels were determined by real-time PCR; *n* = 5, ^∗∗^*p* < 0.01, ^∗∗∗^*p* < 0.001 versus the NC group. (d, e) Pyroptotic proteins were determined by western blotting, and statistical results were obtained by densitometric analysis and normalization to GAPDH; *n* = 5, ^∗∗∗^*p* < 0.001.

Transfection of siRNA was used to knockdown p53, and the results showed that the efficiency of p53 knockdown was feasible (Figures 3(a) and 3(b)). Pyroptotic proteins, including NLRP3, ASC, and cleaved caspase-1, were decreased compared with the negative control (NC) group (Figures 3(c)–3(e)). Additionally, the direct interaction between p53 and NLRP3 was determined by chromatin immunoprecipitation (CHIP) analysis ([Supplementary-material supplementary-material-1]). Taken together, p53 could be considered as a direct regulator of pyroptosis in NSCLC cells.

### 3.3. p53 Inhibits NSCLC Cell Proliferation through Pyroptosis

**Figure 4:**
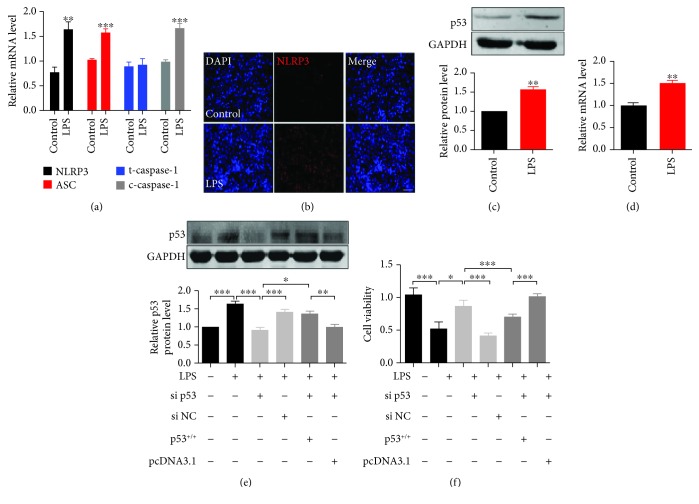
p53-activated pyroptosis suppresses A549 lung cancer cell proliferation. (a) Pyroptotic factor (NLRP3, ASC, caspase-1, and cleaved caspase-1) mRNA levels were determined by real-time PCR; *n* = 5, ^∗∗^*p* < 0.01, ^∗∗∗^*p* < 0.001. (b) Immunofluorescent staining showed the effect of LPS on NLRP3-formed inflammasomes (red) in A549 lung cancer cells. Cell nuclei were visualized by DAPI (blue). Scale bar equals 10 *μ*m. (c, d) p53 protein and mRNA levels were determined by western blotting and real-time PCR, respectively; *n* = 3, ^∗∗^*p* < 0.01. (e) p53 protein levels were determined by western blotting, *n* = 3, normalized to GAPDH, ^∗^*p* < 0.05, ^∗∗^*p* < 0.01, ^∗∗∗^*p* < 0.001. (f) Cell viability was detected by CCK-8 assay in A549 cells; *n* = 10, ^∗^*p* < 0.05, ^∗∗∗^*p* < 0.001.

In order to delineate the function of p53/pyroptosis in NSCLC tumorigenesis, we further gain insight into the effect of p53 on A549 lung cancer cell proliferation. LPS is a normal component of the cell wall of most Gram-negative bacteria, which can trigger cytokine synthesis, secretion, and a subsequent inflammatory process [17]. Thus, we treated A549 cells with LPS to induce pyroptosis. Consistent with previous studies, LPS treatment rapidly and significantly activated pyroptotic effectors (Figure 4(a)). As the sensor of pyroptosis, NLRP3-formed inflammasomes significantly increased in response to LPS stimuli (Figure 4(b)). In addition, LPS remarkably increased p53 levels (Figures 4(c) and 4(d)) and reduced cell proliferation, while knockdown p53 neutralized the effect of LPS on A549 cell proliferation. Oppositely, overexpression of p53 aggravated the reduction of cell viability (Figures 4(e) and 4(f)), and knockdown of pyroptotic proteins NLRP3 in A549 cells blocked LPS-induced pyroptosis or p53 overexpression ([Supplementary-material supplementary-material-1]). Taken together, these results indicated that p53-induced pyroptosis suppressed A549 lung cancer cell proliferation, suggesting its potential on tumor growth inhibition.

### 3.4. p53-Induced Pyroptosis Inhibits Tumor Growth in the Progression of NSCLC

**Figure 5:**
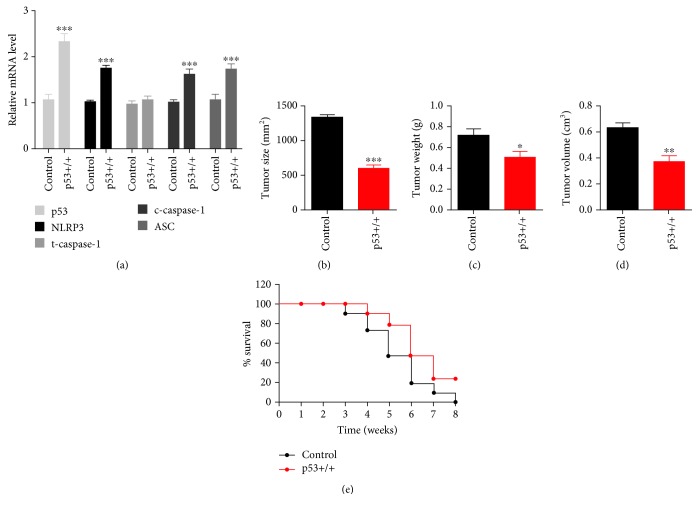
p53-activated pyroptosis inhibits the development of NSCLC in tumor-bearing mice. (a) mRNA levels were measured in the control and p53 overexpression groups. Control mice were transplanted with A549 cells transfected with pcDNA3.1 vector. The p53 overexpression group was induced with transplantation with A549 cells transfected with p53 overexpression vector. Tumor tissues were extracted after mice death or 8 weeks of feeding; *n* = 5 in each group. The data are presented as the mean ± S. E. M.; ^∗∗∗^*p* < 0.001 versus the control group. (b–d) Tumor size, weight, and volume were measured in each group; *n* = 5, ^∗^*p* < 0.05, ^∗∗^*p* < 0.01, ^∗∗∗^*p* < 0.001. (e) Survival percentage was calculated throughout 8-week feeding.

To further determine the effect of p53-induced pyroptosis on tumor growth during NSCLC, we used *in situ* implantation tumor mice of non-small-cell lung cancer to analyze their tumor tissue index and survival rate. Tumor-bearing mice were sacrificed for tumor tissue extraction following 8 weeks of feeding. Pyroptosis and p53 levels were then determined (Figure 5(a)). The result showed that overexpression of p53 significantly increased pyroptosis. Additionally, p53 overexpression in tumor showed a remarkable reduction of tumor size, weight, and volume (Figures 5(b)–5(d)), together with high survival rate compared with controls. These results suggested that overexpression of p53 in NSCLC tumor tissues inhibits the development of NSCLC via activation of pyroptosis. Taken together, p53 activates pyroptosis in NSCLC cells, exerting inhibitory effects on NSCLC tumor growth.

## 4. Discussion

Lung cancer is one of the most life-threatening diseases with the highest mortality and morbidity among multiple types of malignant tumor. NSCLC accounts for nearly 80% of lung cancer patients, and the number of NSCLC patients ascends dramatically [18]. With varied etiology, atmospheric contamination is the main cause to induce the occurrence of NSCLC [19, 20]. It is inevitable that the inferior air quality as well as other pathogenic factors constitutes a risk of high morbidity level [21]. Thus, researches related to improve NSCLC symptoms require further investigation.

As a heterocomposition in an organism, tumor cells are responsible for the activation of immunoreactions, including inflammatory response [22]. Proinflammatory mediators are secreted and released to lead cancer cell inactivation or death in the progression of NSCLC. Inflammatory response includes inflammasome formation and caspase activation. Chronic inflammation is an essential regulator in the process of carcinogenesis via inflammatory cytokines, leading an inflammatory microenvironment. Emerging evidence indicates that chronic inflammation plays an important role at all stages of tumor development, including initiation, growth, invasion, and metastasis [23]. Inflammasomes exert an important function in releasing interleukin- (IL-) 1*β* and IL-18, which in turn prompt inflammatory response to clean up cancer cells [24, 25]. Thi and Hong reported that inflammasome formation is considered as a therapeutic target towards cancer prevention and treatment [26]. Kantono and Guo have illuminated the role of inflammasome formation in the development of colon cancer and breast cancer [27]. Recent studies have reported that pyroptosis, a newly revealed programmed cell death, is pertinent to inflammasome formation, as well as caspase-1 or caspase-4/7/11 activation, enhancing inflammatory reactions. It is undeniable that pyroptosis is tightly related to the progression of cancer and may contribute to inhibitory effect on tumor growth. However, the effect on NSCLC remains to be further clarified.

Programmed cell death was first reported in 1842, which is recognized as an irreversible process involving complex molecular factors or pathways to maintain cellular homeostasis and control diseases in multicellular organisms [28]. Among several programmed cell deaths, pyroptosis is the most recently investigated which is characterized as an inflammatory form of regulated cell death which is triggered by microbial infection, heart attack, stroke, and cancer progression [29]. Pizato et al. have reported that pyroptosis is tightly related to breast cancer cell death and further contributes to improved treatment during breast cancer [30]. However, the relationship between pyroptosis and NSCLC as well as the underlying mechanism remains to be investigated.

Transcription factor p53 is considered to regulate cell cycle and inhibit cell carcinogenesis [31]. However, the mutation of p53 not only loses the function of tumor suppressor but also regains the function of oncogene to promote malignant transformation of cells [32]. Among several noncanonical cell deaths, including apoptosis, ferroptosis, necroptosis, and paraptosis, p53 is the most important regulator to switch these cell deaths [10]. In addition, it is reported that p53 is tightly related to the occurrence and progression of NSCLC [11]; however, the underlying mechanisms remain to be further clarified. Pyroptosis morphologically and mechanistically differs from other types of cell death [33]. Only a few studies show the potential association of p53 with pyroptosis. The key role of p53 on pyroptosis requires a further and comprehensive investigation.

We highlighted three observations in this study. First, we revealed the positive correlation between p53 and pyroptosis in NSCLC tumor tissues. Second, a significant alternation of pyroptosis was found in p53 overexpression or knockdown A549 lung cancer cells, suggesting the regulatory role of p53 on pyroptosis. Third, p53-induced pyroptosis significantly suppresses tumor growth and retards the development of NSCLC, at least in part, improving the symptoms and survival in NSCLC.

Additionally, our research proposed the following consideration: whether specifically activated p53 expression in NSCLC tumor via gene engineering tools could ameliorate mortality and morbidity of NSCLC, or other cancers, providing more beneficial effects to improve the outcomes.

## 5. Conclusions

In this study, we found that p53-induced pyroptosis inhibits tumor growth during NSCLC. Our findings suggest that p53 could be recognized as a potential target to improve NSCLC by the activation of pyroptosis.

## Figures and Tables

**Table 1:** Correlation coefficients of Pearson, Spearman, and Kendall analyses.

	Pearson		Spearman		Kendall	
	*R* value	*p*	*R* value	*p*	*R* value	*p*
NLRP3	0.951	<0.01	0.754	<0.01	0.645	<0.01
ASC	0.974	<0.01	0.935	<0.01	0.825	<0.01
Caspase-1	-0.143	>0.05	-0.114	>0.05	-0.08	>0.05
c-caspase-1	0.975	<0.01	0.880	<0.01	0.751	<0.01

Spearman, Pearson, Kendall, and linear correlation coefficients of p53 and pyroptotic levels in non-small-cell lung cancer (NSCLC) tissues; *n* = 10 in each group.

## Data Availability

No data were used to support this study.

## Abbreviations

NSCLC: Non-small-cell lung cancer
LPS: Lipopolysaccharide
NLRP3: Nucleotide-binding domain and leucine-rich repeat-containing 3
ASC: Apoptosis-associated speck-like protein containing CARD
NC: Negative control
CHIP: Chromatin immunoprecipitation.

## Supplementary Materials

Supplementary Materials Figure S1: p53 protein level is positively related to pyroptosis in tumor tissue of NSCLC patients. mRNA levels of NLRP3, ASC, caspase-1, and cleaved caspase-1 were detected by real-time PCR and analyzed by SPSS. Wild-type p53 protein level shows a positive correlation with NLRP3, ASC, and cleaved caspase-1 in tumor tissues of 20 NSCLC patients. Figure S2: activation of p53 elevates pyroptotic level in A549 NSCLC cells. (a) Activity of p53 was determined by the p53 activity assay kit. A549 lung cancer cells were treated with or without Tenovin-6; *n* = 5, ^∗∗^*p* < 0.01. (b, c) p21 level was determined by real-time PCR and western blotting analysis; *n* = 5, ^∗^*p* < 0.05, ^∗∗^*p* < 0.01. (d) Pyroptotic factor mRNA levels were determined by real-time PCR; *n* = 5, ^∗∗^*p* < 0.01, ^∗∗∗^*p* < 0.001 versus the negative control group. (e, f) Pyroptotic proteins were determined by western blotting, represented images were shown on the left, and statistical results were shown on the right; *n* = 5, ^∗∗^*p* < 0.01, ^∗∗∗^*p* < 0.001. Figure S3: p53 directly regulates pyroptosis by NLRP3. Chromatin immunoprecipitation (CHIP) was used to determine the involvement of p53 in the pyroptotic network. A549 cells were treated with LPS and then collected for CHIP analysis. Figure S4: p53-induced pyroptosis inhibits A549 cell viability. Cell viability was detected by the CCK-8 assay in A549 cells; *n* = 5, ^∗^*p* < 0.05, ^∗∗∗^*p* < 0.001.

## References

[1] Sheng B., Qi C., Liu B., Lin Y., Fu T., Zeng Q. (2017). Increased HSP27 correlates with malignant biological behavior of non-small cell lung cancer and predicts patient’s survival. *Scientific Reports*.

[2] Kanzaki R., Naito H., Kise K. (2017). Gas6 derived from cancer-associated fibroblasts promotes migration of Axl-expressing lung cancer cells during chemotherapy. *Scientific Reports*.

[3] Elmore S. (2007). Apoptosis: a review of programmed cell death. *Toxicologic Pathology*.

[4] Ju Y., Su Y., Chen Q. (2019). Protective effects of Astragaloside IV on endoplasmic reticulum stress-induced renal tubular epithelial cells apoptosis in type 2 diabetic nephropathy rats. *Biomedicine & Pharmacotherapy*.

[5] Prokhorova E. A., Kopeina G. S., Lavrik I. N., Zhivotovsky B. (2018). Apoptosis regulation by subcellular relocation of caspases. *Scientific Reports*.

[6] Zhang Q. L., Yang J. J., Zhang H. S. (2019). Carvedilol (CAR) combined with carnosic acid (CAA) attenuates doxorubicin-induced cardiotoxicity by suppressing excessive oxidative stress, inflammation, apoptosis and autophagy. *Biomedicine & Pharmacotherapy*.

[7] Wang F., Zhang W., Wu T., Chu H. (2018). Reduced interleukin-38 in non-small cell lung cancer is associated with tumour progression. *Open Biology*.

[8] Awais R., Spiller D. G., White M. R. H., Paraoan L. (2016). p63 is required beside p53 for PERP-mediated apoptosis in uveal melanoma. *British Journal of Cancer*.

[9] Hong B., Heuvel A., Prabhu V., Zhang S., el-Deiry W. (2014). Targeting tumor suppressor p53 for cancer therapy: strategies, challenges and opportunities. *Current Drug Targets*.

[10] Ranjan A., Iwakuma T. (2016). Non-canonical cell death induced by p53. *International Journal of Molecular Sciences*.

[11] Kim D. S., Lee W. K., Park J. Y. (2018). Promoter methylation of Wrap53*α*, an antisense transcript of p53, is associated with the poor prognosis of patients with non‑small cell lung cancer. *Oncology Letters*.

[12] Shen N., Li X., Zhou T. (2014). Shensong Yangxin Capsule prevents diabetic myocardial fibrosis by inhibiting TGF-*β*1/Smad signaling. *Journal of Ethnopharmacology*.

[13] Xiao D., Zhou T., Fu Y. (2018). MicroRNA-17 impairs glucose metabolism in insulin-resistant skeletal muscle via repressing glucose transporter 4 expression. *European Journal of Pharmacology*.

[14] Li X., du N., Zhang Q. (2014). MicroRNA-30d regulates cardiomyocyte pyroptosis by directly targeting foxo3a in diabetic cardiomyopathy. *Cell Death & Disease*.

[15] Chu F. I., Marin-Franch I., Ramezani K., Racette L. (2018). Associations between structure and function are different in healthy and glaucomatous eyes. *PLoS One*.

[16] Sun W., Zeng C., Liu S. (2018). Ageratina adenophora induces mice hepatotoxicity via ROS-NLRP3-mediated pyroptosis. *Scientific Reports*.

[17] Li D., Ren W., Jiang Z., Zhu L. (2018). Regulation of the NLRP3 inflammasome and macrophage pyroptosis by the p38 MAPK signaling pathway in a mouse model of acute lung injury. *Molecular Medicine Reports*.

[18] Zhou Y., Ma Y., Shi H., Du Y., Huang Y. (2018). Epidermal growth factor receptor T790M mutations in non-small cell lung cancer (NSCLC) of Yunnan in southwestern China. *Scientific Reports*.

[19] Decoster L., Schallier D. (2019). Treatment of older patients with advanced non-small cell lung cancer: a challenge. *Journal of Geriatric Oncology*.

[20] DeLisser H. M., Keirns C. C., Clinton E. A., Margolis M. L. (2009). “The air got to it:” exploring a belief about surgery for lung cancer. *Journal of the National Medical Association*.

[21] Xie W. Y., Zhou X. D., Yang J., Chen L. X., Ran D. H. (2016). Inhibition of autophagy enhances heat-induced apoptosis in human non-small cell lung cancer cells through ER stress pathways. *Archives of Biochemistry and Biophysics*.

[22] DeCotiis C., Hu Y., Greenberg A. K. (2016). Inflammatory cytokines and non-small cell lung cancer in a CT-scan screening cohort: background review of the literature. *Cancer Biomarkers*.

[23] Rivas-Fuentes S., Salgado-Aguayo A., Pertuz Belloso S., Gorocica Rosete P., Alvarado-Vasquez N., Aquino-Jarquin G. (2015). Role of chemokines in non-small cell lung cancer: angiogenesis and inflammation. *Journal of Cancer*.

[24] Nguyen A. H., Berim I. G., Agrawal D. K. (2015). Chronic inflammation and cancer: emerging roles of triggering receptors expressed on myeloid cells. *Expert Review of Clinical Immunology*.

[25] Ceelen J. J. M., Langen R. C. J., Schols A. M. W. J. (2014). Systemic inflammation in chronic obstructive pulmonary disease and lung cancer. *Current Opinion in Supportive and Palliative Care*.

[26] Thi H. T. H., Hong S. (2017). Inflammasome as a therapeutic target for cancer prevention and treatment. *Journal of Cancer Prevention*.

[27] Kantono M., Guo B. (2017). Inflammasomes and cancer: the dynamic role of the inflammasome in tumor development. *Frontiers in Immunology*.

[28] Clarke P. G., Clarke S. (2012). Nineteenth century research on cell death. *Experimental Oncology*.

[29] Zhaolin Z., Jiaojiao C., Peng W. (2018). OxLDL induces vascular endothelial cell pyroptosis through miR-125a-5p/TET2 pathway. *Journal of Cellular Physiology*.

[30] Pizato N., Luzete B. C., Kiffer L. F. M. V. (2018). Omega-3 docosahexaenoic acid induces pyroptosis cell death in triple-negative breast cancer cells. *Scientific Reports*.

[31] Yang L., Zhou J., Meng F. (2019). G1 phase cell cycle arrest in NSCLC in response to LZ-106, an analog of enoxacin, is orchestrated through ROS overproduction in a P53-dependent manner. *Carcinogenesis*.

[32] Binayke A., Mishra S., Suman P., Das S., Chander H. (2019). Awakening the “guardian of genome”: reactivation of mutant p53. *Cancer Chemotherapy and Pharmacology*.

[33] Yuan Y. Y., Xie K. X., Wang S. L., Yuan L. W. (2018). Inflammatory caspase-related pyroptosis: mechanism, regulation and therapeutic potential for inflammatory bowel disease. *Gastroenterology Report*.

