# Supplementary material for: Transcription Factor p53 Suppresses Tumor Growth by Prompting Pyroptosis in Non-Small-Cell Lung Cancer
Source: Oxid Med Cell Longev. 2019 Oct 13;2019:8746895. doi: 10.1155/2019/8746895 (PMC6815571; doi:10.1155/2019/8746895)
Supplement: Supplementary Materials — Figure S1: p53 protein level is positively related to pyroptosis in tumor tissue of NSCLC patients. mRNA levels of NLRP3, ASC, caspase-1, and cleaved caspase-1 were detected by real-time PCR and analyzed by SPSS. Wild-type p53 protein level shows a positive correlation with NLRP3, ASC, and cleaved caspase-1 in tumor tissues of 20 NSCLC patients. Figure S2: activation of p53 elevates pyroptotic level in A549 NSCLC cells. (a) Activity of p53 was determined by the p53 activity assay kit. A549 lung cancer cells were treated with or without Tenovin-6; n = 5, ∗∗p < 0.01. (b, c) p21 level was determined by real-time PCR and western blotting analysis; n = 5, ∗p < 0.05, ∗∗p < 0.01. (d) Pyroptotic factor mRNA levels were determined by real-time PCR; n = 5, ∗∗p < 0.01, ∗∗∗p < 0.001 versus the negative control group. (e, f) Pyroptotic proteins were determined by western blotting, represented images were shown on the left, and statistical results were shown on the right; n = 5, ∗∗p < 0.01, ∗∗∗p < 0.001. Figure S3: p53 directly regulates pyroptosis by NLRP3. Chromatin immunoprecipitation (CHIP) was used to determine the involvement of p53 in the pyroptotic network. A549 cells were treated with LPS and then collected for CHIP analysis. Figure S4: p53-induced pyroptosis inhibits A549 cell viability. Cell viability was detected by the CCK-8 assay in A549 cells; n = 5, ∗p < 0.05, ∗∗∗p < 0.001. [file 8746895.f1.docx]

**Transcription factor p53 suppresses tumor growth by prompting pyroptosis**

**in non-small cell lung cancer**

Tianze Zhang^1^, Yongchao Li^1^, Ruidong Zhu^1^, Pengcheng Song^1^, Youlei Wei^1^, Tian Liang^2^, Guangquan Xu^1^*

^1^ Department of Thoracic Surgery, the 2nd Affiliated Hospital of Harbin Medical University, Harbin, China, 150081

^2^ Department of obstetrics and gynaecology, the 1st Affiliated Hospital of Harbin Medical University, Harbin, China, 150081

*Corresponding authors

Correspondence to Guangquan Xu, PhD, Professor, Department of Thoracic Surgery, the 2nd Affiliated Hospital of Harbin Medical University, Harbin, China, 150086

Tel: 86 13904503643

Email: 13904503643@163.com

**Supplementary results**

**
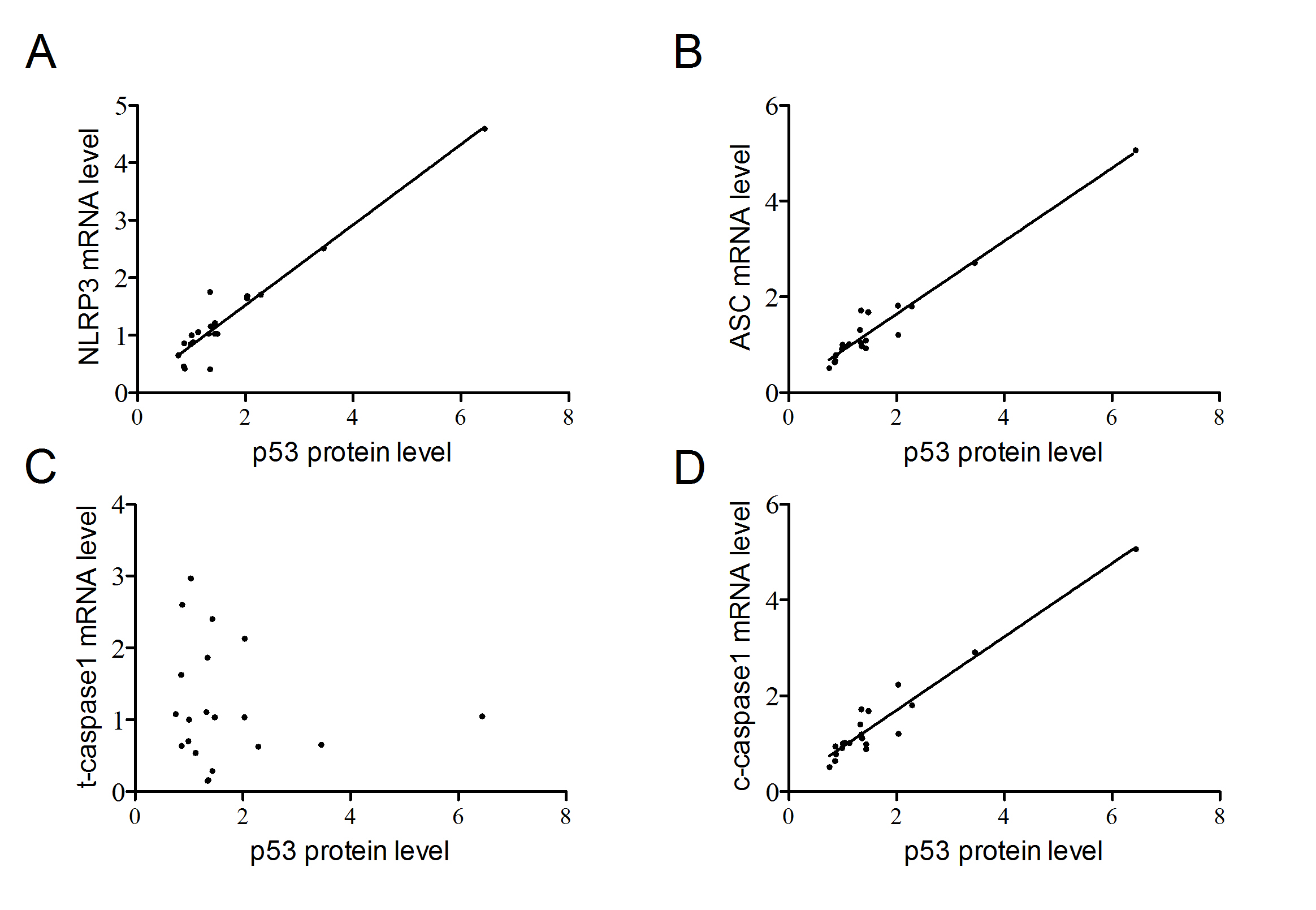
**

**Figure S1. p53 protein level is positively related to pyroptosis in tumor tissue of NSCLC patients.** mRNA levels of NLRP3, ASC, caspase1 and cleaved-caspase 1 were detected by real-time PCR and analyzed by SPSS. Wild type p53 protein level shows a positive correlation with NLRP3, ASC and cleaved caspase1 in tumor tissues of 20 NSCLC patients.

**
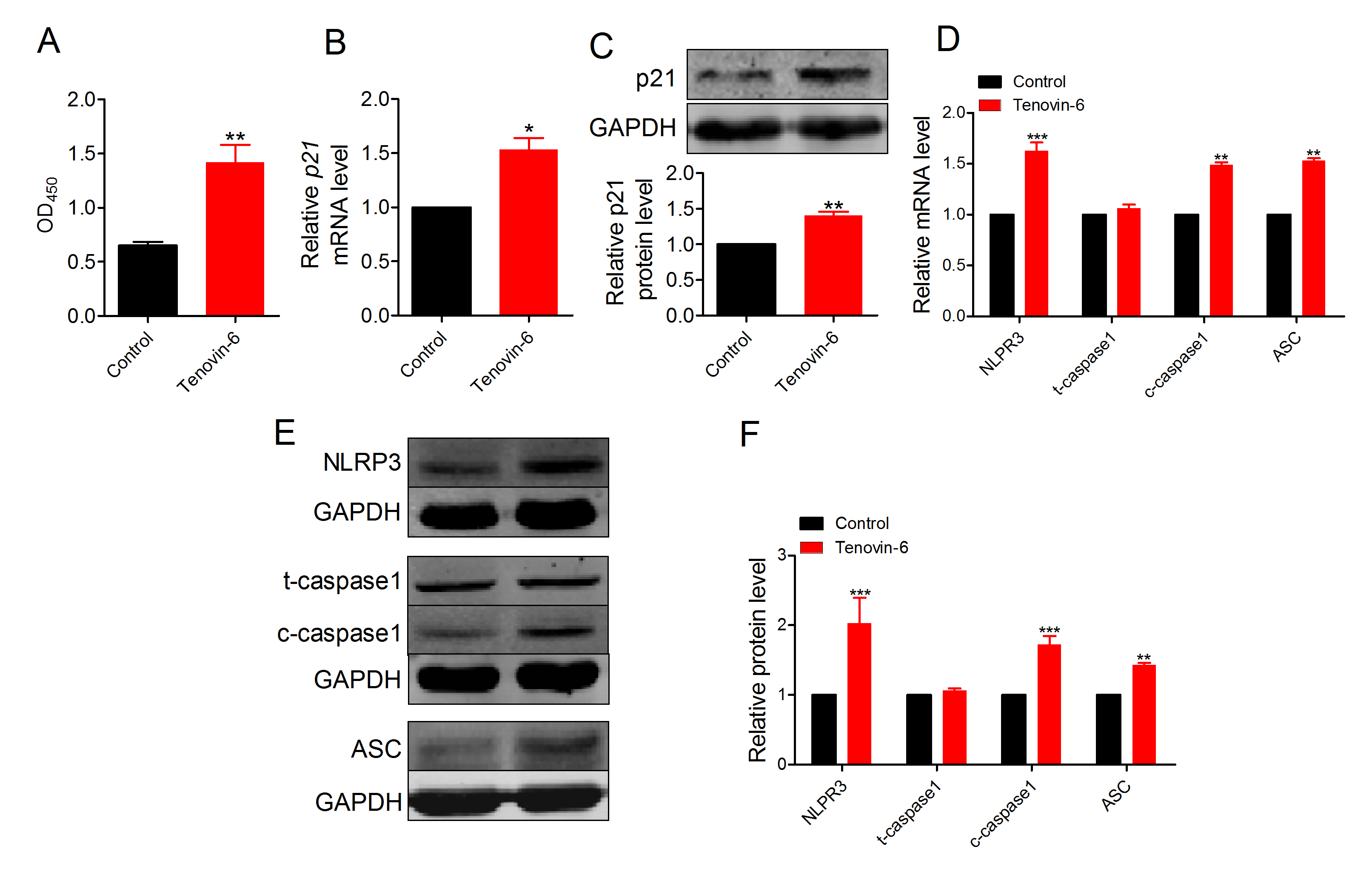
**

**Figure S2. Activation of p53 elevates pyroptotic level in A549 NSCLC cells.** (A) Activity of p53 was determined by p53 activity assay kit. A549 lung cancer cells were treated with or without Tenovin-6, n=5, ***p*<0.01. (B, C) p21 level was determined by real-time PCR and western blotting analysis, n=5, **p*<0.05, ***p*<0.01. (D) Pyroptotic factors mRNA levels were determined by real-time PCR, n=5, ***p*<0.01, ****p*<0.001 versus negative control group. (E, F) Pyroptotic proteins were determined by western blotting, represented images were showed on the left, and statistic result were showed on the right, n=5, ***p*<0.01, ****p*<0.001.

**
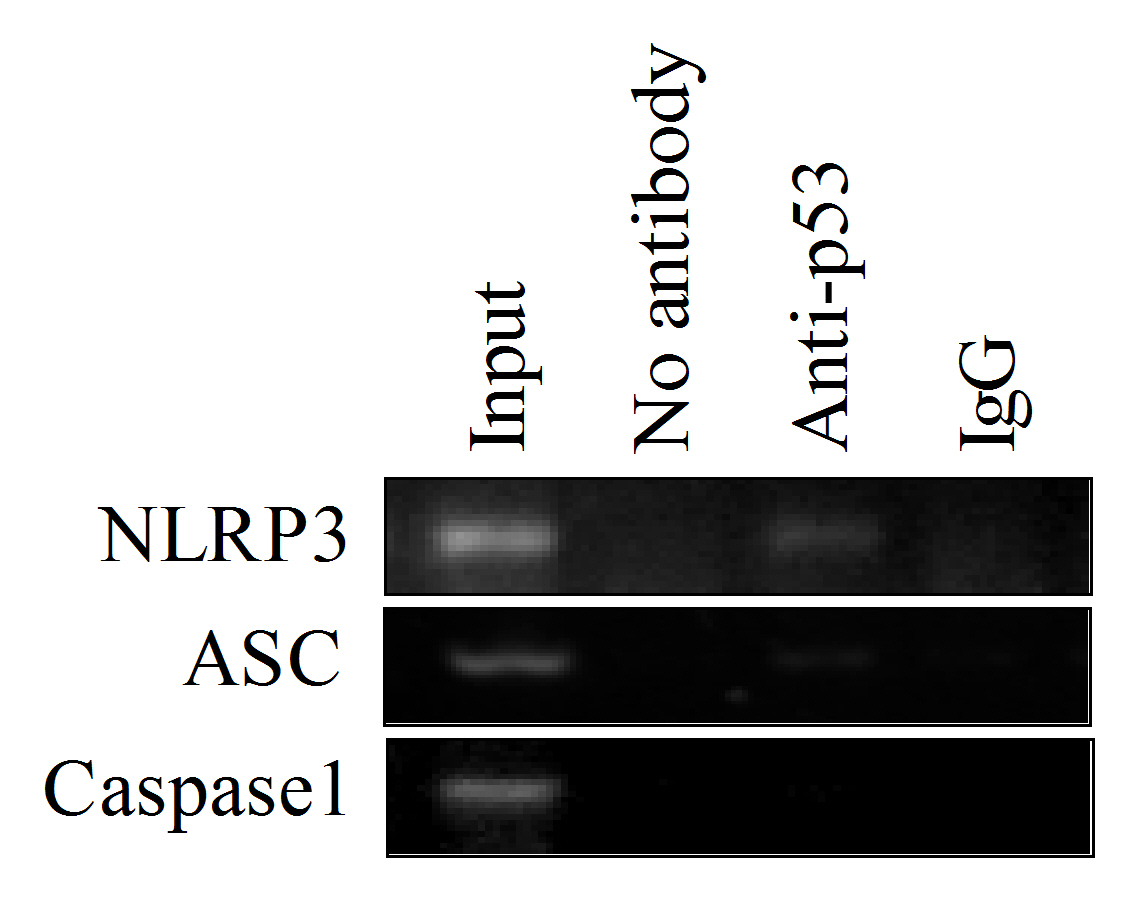
**

**Figure S3. p53 directly regulate pyroptosis by NLRP3.** Chromatin Immunoprecipitation (CHIP) was used to determine the involvement of p53 in pyroptotic network. A549 cells were treated with LPS and then collected for ChIP analysis.

**
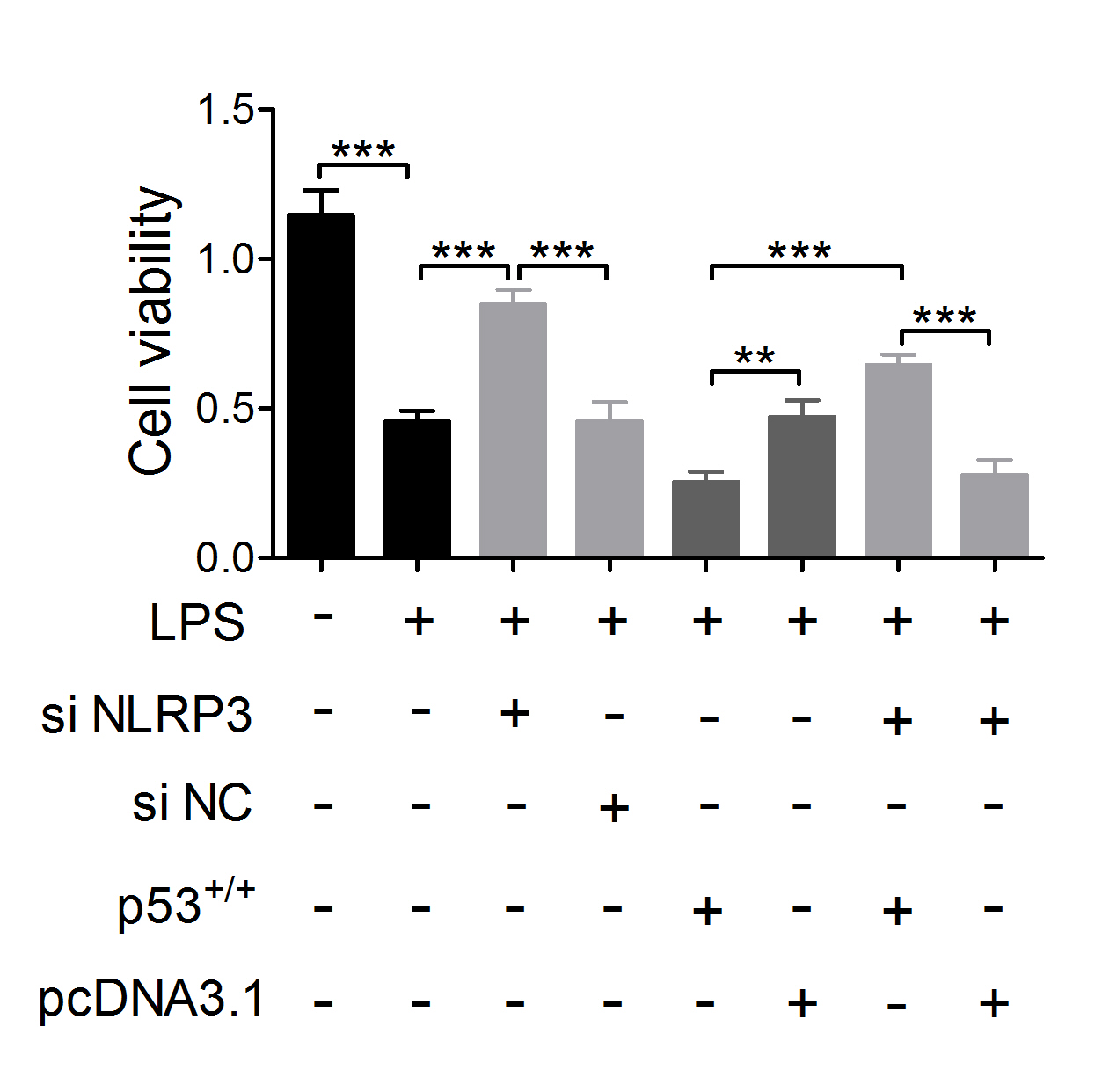
**

**Figure S4. p53-induced pyroptosis inhibits A549 cell viability.** Cell viability was detected by CCK-8 assay in A549 cells, n=5, **p*<0.05, ****p*<0.001.
